# The source and fate of *Mycobacterium tuberculosis* complex in wastewater and possible routes of transmission

**DOI:** 10.1186/s12889-022-12527-z

**Published:** 2022-01-20

**Authors:** Hlengiwe N. Mtetwa, Isaac D. Amoah, Sheena Kumari, Faizal Bux, Poovendhree Reddy

**Affiliations:** 1 Department of Community Health Studies, Faculty of Health Sciences, Durban University of Technology, PO Box 1334, Durban, 4000 South Africa; 2 Institute for Water and Wastewater Technology (IWWT), Durban University of Technology, PO Box 1334, Durban, 4000 South Africa

**Keywords:** *Mycobacterium tuberculosis* complex (MTBC), Wastewater, Sewage, Environment

## Abstract

**Background:**

The *Mycobacterium tuberculosis* complex (MTBC) consists of causative agents of both human and animal tuberculosis and is responsible for over 10 million annual infections globally. Infections occur mainly through airborne transmission, however, there are possible indirect transmissions through a faecal-oral route which is poorly reported. This faecal-oral transmission could be through the occurrence of the microbe in environments such as wastewater. This manuscript, therefore, reviews the source and fate of MTBC in the wastewater environment, including the current methods in use and the possible risks of infections.

**Results:**

The reviewed literature indicates that about 20% of patients with pulmonary TB may have extra-pulmonary manifestations such as GITB, resulting in shedding in feaces and urine. This could potentially be the reason for the detection of MTBC in wastewater. MTBC concentrations of up to 5.5 × 10^5^ (±3.9 × 10^5^) copies/L of untreated wastewater have been reported. Studies have indicated that wastewater may provide these bacteria with the required nutrients for their growth and could potentially result in environmental transmission. However, 98.6 (± 2.7) %, removal during wastewater treatment, through physical-chemical decantation (primary treatment) and biofiltration (secondary treatment) has been reported. Despite these reports, several studies observed the presence of MTBC in treated wastewater via both culture-dependent and molecular techniques.

**Conclusion:**

The detection of viable MTBC cells in either treated or untreated wastewater, highlights the potential risks of infection for wastewater workers and communities close to these wastewater treatment plants. The generation of aerosols during wastewater treatment could be the main route of transmission. Additionally, direct exposure to the wastewater containing MTBC could potentially contribute to indirect transmissions which may lead to pulmonary or extra-pulmonary infections. This calls for the implementation of risk reduction measures aimed at protecting the exposed populations.

## Background

Tuberculosis (TB) is a communicable disease and one of the top ten causes of death globally, ranking above human immunodeficiency virus/ acquired immunodeficiency syndrome (HIV/AIDS) [1, 2]. It is caused by a group of closely related slowly growing mycobacteria, collectively named *Mycobacterium tuberculosis* complex (MTBC), which infect a large spectrum of mammals, including humans [3, 4]. This includes *M. bovis*, the causative agent of tuberculosis in both animals and humans [5–8] and *M. africanum*, the causative agent of human tuberculosis (mainly in Western Africa [9, 10]. Lesser-known members of this group are *M. microti*, *M. caprae, M. pinnipedii*, *M. canetti* and *M. mungi,* usually associated with infections animals with possible transmission to humans. According to the World Health Organisation (WHO), an estimated 10 million people (as of 2018) were infected with TB worldwide [11]. Geographically, Africa accounted for 24% of the reported TB cases in 2018 [2]. Human immunodeficiency virus (HIV) is considered an important risk factor for contracting TB in most African countries especially South Africa, with co-infection associated with increased morbidity and mortality [12–14]. Over 70% (6 million) of humans co-infected with TB and HIV/AIDS live in sub-Saharan Africa where bovine TB represents a potential health hazard to humans as well [15, 16].

The main infection route for TB has been reported to be through exposure to aerosols from infectious patients [17–22]. This fundamentally shows the airborne transmission of pulmonary TB and is currently widely accepted as the primary mechanistic transmission route [23, 24]. Although airborne transmission is the main route for TB, other routes have been reported. For example, in 1905, Calmette and Guérin postulated that TB could be transmitted through contaminated food [24, 25]. Gao et al. [26] also provided evidence that guinea pigs could be infected by drinking MTBC contaminated water. The clinical and pathological observations in the infected animals were similar to those found in guinea pigs infected via the respiratory or subcutaneous routes. This shows the possible oral transmission of TB in exposed individuals.

The environmental occurrence of pathogenic mycobacteria has received less attention in comparison to its occurrence in clinical settings. Nevertheless, there is a growing body of evidence to show that water could be a significant vehicle for the transmission of these organisms [27, 28]. Previous studies revealed that environmental contamination, from faecal shedding, provided the potential and indirect routes for transmission of *M. bovis* infection [14, 29]. The shedding of *M. bovis* cells has already been demonstrated in many animals via oro- nasal mucus, sputum, urine, feaces and wound discharges [25, 30–32]. Investigating this type of indirect transmission is challenging because it results at least from the combination of three essential factors i.e., i) the environmental contamination by shedding from infected animals, ii) the persistence of the bacteria under a viable state in environmental matrices and finally iii) the interaction between a new susceptible host with the contaminated matrices [25]. This route of transmission has been implicated most frequently in zoonotic infections than human-to-human infections [14].

Wastewater serves as a link between human activities and the environment and could be the first medium that may be contaminated with MTBC via faecal shedding. However, studies on the occurrence of MTBC in different environmental matrices has not received priority, therefore there’s a lack of proper detection techniques for MTBC in the environment. The study of *M. tuberculosis* in wastewater could potentially address limitations in our understanding of transmission, which is currently achieved almost exclusively through studying clinical samples. Therefore, this review systematically summarized the current knowledge on the occurrence of MTBC in wastewater. The current methods of detection and the risk of infections due to exposure to wastewater contaminated with these pathogens are also discussed.

## Methodology

### Literature search strategy

All the papers reviewed were taken from the sources that are available publicly. Publications of potential interest were retrieved from these databases, Google Scholar, Web of Science, Science Direct, and PubMed. The keywords and word strings used were, tuberculosis OR *Mycobacteria* OR *Mycobacterium tuberculosis* OR *Mycobacterium tuberculosis* complex OR *Mycobacterium bovis* AND Wastewater OR Sewage OR Water. Only papers written in the English language were reviewed with no limitation on the year of publication and geographical location of studies. After searching each database, individual article titles and abstracts were assessed to determine their relevance to the scope of this review. Three categories of empirical studies were included in the review: detection of mycobacteria or *Mycobacterium tuberculosis* complex in wastewater and the environment, public health risks from the exposure to wastewater or water contaminated by wastewater (specifically to sewage workers, health care workers and the nearby community), health risks of wastewater irrigation, indirectly health risks, and studies on contamination of crops used for human consumption. Studies that included soil or wastewater contamination by members of the *Mycobacterium tuberculosis* complex were also included in the review.

### Data extraction

Relevant data extracted included authorship, year of publication, location of study, isolated bacteria, sample matrix, and finally the results obtained. The results obtained included MTBC species, survival period in the environment, source of the pathogenic bacteria, genetic epidemiology of pathogenic bacteria detected. The retrieved information was reviewed and presented in different sections focusing on, the source of the MTBC in human excreta (Section 3), the occurrence and fate of these MTBC cells in the wastewater environment (Section 4) and an assessment of the potential risks associated with MTBC occurrence in wastewater (Section 5).

## Source of MTBC cells in excreta

The occurrence of MTBC cells in wastewater could be a result of the shedding of these cells in excreta and other bodily fluids from infected individuals. This section reviews the available literature on gastrointestinal infections associated with TB (GITB) that may result in excretion in feaces and reports of the actual detection of these cells in excreta.

### Gastrointestinal (GI) infections with MTBC

The primary site of TB is usually the lungs, from which it can get disseminated into other parts of the body [33]. The other routes of spread can be contiguous involvement from adjacent tuberculous lymphadenopathy or primary involvement of extrapulmonary organs [34–36]. It is estimated that close to 20% of patients with pulmonary TB may have extra-pulmonary manifestations such as GITB [37, 38]. GITB is usually caused primarily through ingestion of the pathogenic MTBC in water and food (such as non-heat-treated milk, vegetables and meat [39, 40]. Animals ingest relatively more vegetable feed, which is often contaminated with mycobacteria, or their surface is contaminated with soil often containing mycobacteria [40, 41]. The ingestion of water and food contaminated with MTBC is therefore the main route of GITB [42–44]. Other routes include infected sputum, hematogenous spread from distant tubercular focus, contagious spread from infected adjacent foci and through a lymphatic channel [34].

The mucosal layer of the gastrointestinal (GI) tract can be infected with the bacilli with the formation of epithelioid tubercles in the lymphoid tissue of the submucosa [34]. The most common region within the GI tract affected is the ileocaecal region, due to its richness in lymphoid tissue and increased absorption rate [37]. Some of the symptoms of GITB include diarrhoea, nausea and vomiting [45]. Diagnosis of gastrointestinal tuberculosis (GITB) is difficult resulting in increased morbidity [46]. However, GITB infections have been reported over the years by multiple studies [34, 46–52].

### *Mycobacterium tuberculosis* complex in excreta

Most mycobacterial pathogens, causing tuberculosis and tuberculosis-like infections in other soft tissues or lymph nodes, are excreted via human urine if the infection is in kidneys or stool for GITB infections [53–57]. The detection of TB through human stool analysis has also been reported [58]. Mitchell et al. [59] reported that clinical signs of TB infection are often correlated with high shedding levels (above 50 colony forming units (CFU)/gram of faeces). *M. bovis* has been detected in both goat and cattle faeces [14]. Despite being a human pathogen *M. tuberculosis* has been detected in cattle as well [14]. This indicates interspecies infection, which can be determined based on the detection of these different species in faecal samples. For instance, the urine of badgers and possums has been reported to aid in the interspecies transmission of bovine tuberculosis to cattle due to the detection of this pathogen in the urine [40, 60]. Therefore, these reports of MTBC shedding in urine and stool could result in their occurrence in wastewater (discussed below, Section 4).

## Occurrence and fate of *Mycobacterium tuberculosis* complex (MTBC) in wastewater

The occurrence of MTBC in wastewater has been reported over the years as shown in Table 1. These studies detected the presence of MTBC in different matrices, such as wastewater or sewage and surface water impacted by wastewater. The literature on MTBC occurrence in wastewater has focused largely on the molecular-based detection of these cells with less to no quantification data presented. This could be attributed to the challenges with the methods for quantification as described in Section 4.1. The most commonly reported MTBC in wastewater are *M. tuberculosis* and *M. bovis* (See Table 1). These bacteria were commonly reported, using conventional culture-based, biochemical analysis and molecular-based techniques, in raw wastewater [61–64], treated wastewater [64–66], activated sludge [65], soil [67] and water [16, 61, 67]. This could be attributed to the high number of human infections caused by these pathogens [68]. *M. bovis* is the second most commonly reported member of the MTBC based on the number of publications reporting the detection of this bacteria in wastewater. The presence of these pathogens in wastewater could either result in potential infections directly or indirectly through the contamination of drinking water. Suliman et al. [61] reported the occurrence of *M. tuberculosis* in both drinking water and wastewater from a hospital in Pakistan, this could be an example of wastewater contamination of drinking water [69]. In some instances, these wastewater samples or wastewater impacted surface water and soil were taken from areas connected to hospitals [70, 71], households [70–72] and farms [62, 73, 74]. The available information on MTBC detection in wastewater and other environmental matrices shows an early interest in this domain. The first available reports in this area were from the early 1960s [64], subsequently, a reduction in output was observed in the 1980s. In the last decade, there has been an increase in the number of publications on the occurrence of MTBC in wastewater possibly due to advancements in molecular-based detection methods and high-throughput sequencing data (See Table 1).

**Table 1:** Occurrence of MTBC in wastewater

Specific MTBC organism	Sample matrix	Study location	Detection method	Target	Reference
*M. tuberculosis*	Raw sewage, sewage effluent	Poland	Culture-based		[64]
*M. tuberculosis*	Sanatorium sewage: inlet, settling tank and outlet	India	Culture-based		[66]
*M. bovis, M. tuberculosis*	Sewage from cattle farm used for pastures	Poland	Culture-based		[73, 74]
*M. bovis, M. tuberculosis*	Sewage from tuberculous sanatorium and hospitals, towns and sewage purification plants	Poland	Culture-based (Sewer swabs)		[70, 71]
*M. tuberculosis*	Sewage sediment	Poland	Culture-based		[75]
*M. bovis*, *M. tuberculosis*	Sewage water around tuberculous sanatoria	Kazakhstan	Culture-based		[63]
*M. tuberculosis*	Wash-off water from wearing apparel, crockery, household utensils, etc	Russia	Culture-based		[72]
*M. tuberculosis, M. bovis*	River sediment (wastewater present)	Romania, Portugal,	PCR-based	16SRNA sequence	[62, 76]
*M. tuberculosis*	Fresh sewage used for pastures and fields	Germany	Culture-based		[62]
*M. tuberculosis*	Activated sludge and effluent	Hong Kong	PCR-based	16S rRNA gene & IS6110	[63]
*M.bovis/caprae/microti/tuberculosis/africanum/pinnipedii*	River (sediment/ water)	Portugal	PCR-based	16SRNA sequence	[76]
*M. tuberculosis*	soil and water	Tehran, Iran	Culture, biochemical and PCR-based	16S–23S RNA gene spacer polymerase chain reaction	[67]
*M. tuberculosis*	Drinking water and sewage water	Pakistan	Culture, biochemical and PCR-based	RNA converted to cDNA for amplification	[61]
*Mycobacterium tuberculosis complex*	water	South Africa	PCR-based	Genomic DNA	[16]

### Methods used for the detection of MTBC in wastewater

**Fig. 1:**
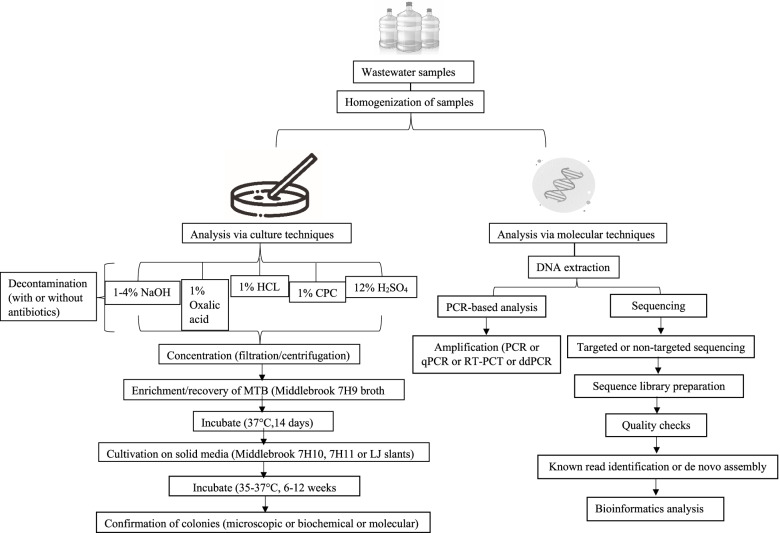
Representation of the common sample-processing framework for the detection of MTBC in wastewater samples

The lack of data on the occurrence of MTBC in environmental matrices could be mainly due to the lack of sensitive and mass-scalable techniques to detect these organisms in environmental samples [24, 76, 77]. Methods for the detection of MTBC in the environment can be categorised into two, culture-based and molecular techniques, these are discussed below and presented in Fig. 1.

#### Culture-based methods for the detection of MTBC in wastewater

Isolation and culturing of MTBC from wastewater require two key steps, disinfection/decontamination of the samples to remove other microorganisms capable of interfering with their (MTBC) growth and concentration of the samples.

Disinfection/decontamination is usually achieved using 1–4% NaOH, 1% Oxalic acid, 1% HCL [78], or 1% Cetylpyridinium chloride (CPC) & 12% sulfuric acid (H_2_SO_4_) [67, 79–82]. All these chemicals exert adverse effects on the growth of other microflora that may be in the sample. Numerous studies have previously recommended CPC as the most suitable chemical for decontamination [30, 80, 82]. This is because the low toxicity of CPC to mycobacteria enables a fast recovery rate of mycobacteria [80, 82].

However, the elimination of nontarget microorganisms by chemical decontamination is insufficient [83]. The incorporation of antimicrobials in the decontamination procedure will remove most of the contaminant bacteria and provide the opportunity for bacilli to grow, which results in highly positive cultures [82, 84]. The use of antibiotics, such as nalidixic acid (NAL), vancomycin (VAN) and amphotericin B (AMB), in previous studies, has shown desired effects by reducing contamination rate and improving culture sensitivity [82]. In addition to inactivating other microorganisms, the disinfection/decontamination agents may also inactivate some of the mycobacteria but to a lesser extent [78, 82, 85]. The lesser impact of these decontamination chemicals could be due to the tough cell wall of these mycobacteria. Therefore it is recommended that the chemical effects should be balanced to support mycobacterial growth and eliminate contaminating microorganisms [82]. Minor inhibitory effects can be ignored because of the significant improvement in the sensitivity of culture due to the use of antibiotics [82, 83].

The next step after decontamination/disinfection is the concentration of the MTBC cells. The most common methods of MTBC concentration from wastewater are filtration (0.2–0.5 um) and centrifugation [86]. After cell concentration, the mycobacterial cells are isolated using specific culture media. Middlebrook 7H9 broth (mostly used for enrichment or recovery of MTBC), 7H10 agar, 7H11 agar or Lowenstein Jensen (L-J) slants are the most commonly used isolation media for mycobacterium, with recommended incubation temperature of 35 °C–37 °C for 6–12 weeks for slow-growing mycobacteria [61, 67, 87]. Solid media may also at times be supplemented with a group of antibiotics such as Polymyxin B, Amphotericin B, Carbenicillin and Trimethoprim (PACT) or Polymyxin-B, Amphotericin-B, Nalidixic acid, Trimethoprim, Azilocillin (PANTA) [88, 89]. Furthermore, malachite green, which is the selective antifungal agent in L–J, shows inhibitory effects on the growth of different mycobacterial species [82].

MTBC has been successfully cultured from environmental samples [67, 77], using the approaches mentioned above. However, limited sensitivity has been observed due to bacterial overgrowth and the presence of “differentially culturable” (or “viable but non-culturable”) MTBC organisms [77, 90, 91]. Many bacteria, including a variety of important human pathogens, are known to respond to various environmental stresses by entry into a novel physiological state, where the cells remain viable but are no longer culturable on standard laboratory media [92–94]. On resuscitation from this ‘viable but non-culturable’ (VBNC) state, the cells regain culturability and the renewed ability to cause infection. In the case of wastewater, some members of MTBC have been reported as amoeba-resistant [95] also detailed in section 4.3.2. Additionally, MTBC in wastewater may enter into the VBNC state in response to stresses such as lack of oxygen, nutrient scarcity, predation (e.g amoeba), chemical stress (chlorine). This could be one of many ways through which these bacteria may be able to survive most wastewater treatment processes in addition to their intrinsic abilities to survive extreme environmental conditions. It is likely that the VBNC state is a survival strategy, although several interesting alternative explanations have been suggested. For example, it appears that the ‘latent’ or the ‘dormant’ phase of *M. tuberculosis* infections represents the VBNC state in this pathogen [93, 96] and that the recurrence of tuberculosis years after a person was thought to be tuberculosis free is due to resuscitation of this pathogen from the VBNC state [92, 97]. As cells in the VBNC state are no longer culturable, alternate nonculture methods must be used to demonstrate that cells in this state are alive. Commonly used are reagents (e.g. the BacLights Live/Dead assay) designed to demonstrate, through direct microscopic examination, the presence of an intact cytoplasmic membrane (e.g. the BacLights Live/Dead assay) [98]. Despite the application of these methods for the detection of these MTBC cells using culture-based techniques, there is no consensus on the method yielding the highest number of mycobacteria.

A study by Suliman et al. [61] reported the detection of MTBC organisms from hospital sewage water and drinking water by conventional culturing techniques, followed by biochemical analysis. Velayati et al. [67] and his colleagues were successful in detecting *M. tuberculosis* from 80% of hospital wastewater samples from different locations [99]. also reported a higher recovery of *M. tuberculosis* from water (86.5%) than soil (13.4%). The majority of *M. tuberculosis* isolates were recovered from raceway systems (56 of 500, 11.2%) or dump water (15 of 200, 7.5%). Three multidrug-resistant *M. tuberculosis* (MDR-TB) (3.6%), four mono drug-resistant strains (three isoniazid and one rifampin, 4.8%), and 58 pan susceptible strains (70%) were also detected among the water and soil isolates.

Some limitations have been identified with the use of culture-based methods for the detection of MTBC in wastewater, per the published literature the main issues are contamination from fast growing bacteria. This may result in overgrowth of these bacteria on culture media, which could out compete the MTBC. Decontamination/disinfection or the use of antibiotics has been introduced as a means to reduce the impact of fast growing bacteria on culture plates, however, as discussed above these decontamination/disinfection techniques could also have a detrimental effect on some of the mycobacteria. Furthermore, MTBC in wastewater could easily enter into the VBNC state which may lead to their non-detection via culturing. This could potentially result in the underestimation of concentrations in wastewater.

#### Molecular methods for the detection of MTBC in wastewater

The development of molecular methods has assisted in addressing some of the challenges associated with the detection of MTBC cells in environmental matrices. While conventional molecular methods (e.g. polymerase chain reaction (PCR)) do not distinguish viable from non-viable organisms, several molecular methods have been developed to do so, including detection of mRNA or selective detection of intracellular markers [100–102]. An increasingly popular molecular method that can be used to detect MTBC cells in the VBNC state is reverse transcriptase (RT)-PCR, which detects RNA. Because the half-life of bacterial mRNA is typically only 3–5 min [103], continued gene transcription by non-culturable cells is considered an excellent indicator of bacterial cell viability. Molecular detection of MTBC has been demonstrated in filtered air samples [104], however, no study to date has applied these molecular techniques to detect MTBC in wastewater samples. This is despite an increasingly robust literature on the detection of various pathogens in natural and built environments [24, 105]. Therefore, there is a knowledge gap in relation to molecular detection of MTBC in wastewater using PCR techniques. The biggest limitation associated with the molecular detection of bacteria in wastewater is the inability to differentiate between live or dead cells. This is a major issue especially in the context of wastewater treatment efficiency and risk of infection assessment. The introduction of RT-PCR has the potential to address this challenge however as mentioned above, this has not been applied yet for the study of MTBC in wastewater.

#### High-throughput sequencing for the detection of MTBC in wastewater

Other genomic or molecular methods such as sequencing have been applied to successfully identify pathogens, study population structure and pathogen evolution among other outcomes [106, 107]. For instance, whole-genome sequencing (WGS) has become the preferential technique for infectious disease epidemiology such as tuberculosis, support for public health and veterinary health professionals in decision making [108–110]. WGS approaches make use of DNA sequencing platforms for the reconstruction of DNA sequences of the genome of an organism [109]. MTBC strains have a single-chromosome genome, which makes these organisms well suited for WGS [111]. The use of WGS for the design and implementation of direct patient treatment and improvement of surveillance systems has been reported in certain countries in relation to *M. tuberculosis* [109, 112].

Irrespective of the sequencing platform, there is a common pathway or workflow, these are, (1) nucleic acid extraction (either DNA or RNA) is first extracted from the samples or isolates, (2) enzymatic processing of extracted nucleic acid, (3) sequencing of multiple fragments of nucleic material in parallel, and (4) finally bioinformatic analyses of data generated from the sequencing [113].

The use of such sequencing approaches for the detection of MTBC organisms in wastewater samples has seen an increased interest in recent years. Some of these reports do not provide species identification, with identification down to only the genus [114–119]. However, others have identified known human pathogens, such as *M. tuberculosis* [120] and animal pathogens, *M. avium* and *M. bovis* [121]. Additionally, other lesser-known species have been identified through this sequencing approach [122, 123]. Table 2 presents some of the publications on the use of different sequencing approaches for the detection of mycobacteria in wastewater and sludge. Therefore, molecular sequencing methods/techniques are useful tools that could potentially play a significant role in the detection of MTBC in wastewater. However, it must be noted that several laboratories do not have access to these sequencing platforms and in some instances, access does not address the issue of costs and skills. This has limited the widespread adoption of these methods. Therefore, there is a need to identify and optimize cost-effective alternative sequencing approaches.

**Table 2:** Detection of MTBC organisms using sequencing approaches

Specific MTBC organism	Sample matrix	Study location	Detection method	Sequencing method	Reference
*Mycobacterium avium,* *Mycobacterium abscessus, Mycobacterium bovis,* *Mycobacterium kansasii, Mycobacterium marinum*	Wastewater	Hong Kong	Illumina HTS	HTS-based metagenomic analysis	[122]
*Mycobacterium* sp., *Mycobacterium fortuitum*	Wastewater and sludge	China	Illumina HiSeq	Metagenomic sequencing	[123]
*Mycobacterium sp*		China	Illumina Hiseq	Paired-end sequencing	[114]
*Mycobacterium* sp	Wastewater	South Africa	16S-rRNA-Based Amplicon Sequencing	Paired-end sequencing	[115]
*Mycobacterium sp*	wastewater	Singapore	Illumina HiSeq2500	Metagenomic sequencing	[117]
*Mycobacterium sp*	wastewater	Vietnam	Illumina TruSeq	Cluster generation and paired-end sequencing	[116]
*Mycobacterium sp*	Biosolids	Colombia	Illumina MiSeq	Metagenomics and 16S-amplicons sequencing	[118]
*Mycobacterium tuberculosis,* *Mycobacterium sp*	wastewater	USA	Illumina MiSeq	Shotgun metagenomic analyses	[121]
*Mycobacterium sp*	wastewater	Taiwan	Illumina HiSeq PE150	Paired-end sequencing	[124]
*Mycobacterium sp*	wastewater	USA	NGS—next-generation sequencing	Shotgun whole genome sequencing	[119]

### Source of *Mycobacterium tuberculosis* complex in wastewater

The occurrence of MTBC in wastewater (Table 1) could be from various sources including domestic, industrial and agricultural.

i) *Domestic wastewater:* This could be primarily due to gastrointestinal infections with MTBC (See Section 3.1) which results in the shedding of MTBC cells in human excreta. The human sewage microbiome is referred to as the collective microbes in sewage from human domestic waste such as feaces, urine, sweat, washing, bathing, etc. [65]. This is mainly derived from the human body including the skin, respiratory tract, oral cavity, gastrointestinal tract, and urogenital tract which ends up in wastewater treatment plants [65, 125]. Wastewater from hospitals and facilities that receive patients infected with contagious microorganisms has dense concentrations of pathogens which include *Mycobacterium* spp. [126]. A study by Jensen [127] demonstrated the occurrence of tubercle bacilli in considerable numbers in the wastewater systems of several towns containing tuberculosis clinics. It is therefore important to note that sewage systems from communities with high TB infections, facilities and institutions receiving pathogen carriers are at risk of contamination due to the presence of these organisms at high concentrations [126, 128].

ii. *Industrial wastewater:* This includes wastewater from slaughterhouses and may constitute the largest source of contamination of the environment in some regions [129–131]. Improper management of abattoir wastes and subsequent disposal either directly or indirectly into river bodies portends serious environmental and health hazards (possible infection from *M. bovis*) both to aquatic life and humans [132, 133]. Irshad et al. [134] reported that improper disposal of wastes from slaughterhouses could lead to the transmission of pathogens to humans and cause zoonotic diseases such as bacillosis, salmonellosis, brucellosis, and helminths. Pokam et al. [135] reported that *M. bovis* can be transmitted by aerosol and ingestion of infected carcasses.

iii. *Wastewater from agricultural fields:* This includes animal excrement, manure and other components: Agricultural fields using manure as a soil amendment could potentially contribute significantly to the pathogen, such as MTBC, in wastewater. The presence of different pathogens in manure has been reported extensively [136–139]. MTBC cells, most especially *M. bovis* have been detected commonly in manure [140–142], this could therefore significantly result in the contamination of water sources with these pathogens. Additionally, the occurrence of these pathogens in manure could potentially result in the infection of both humans and animals. In addition to the manure, the reports of shedding of MTBC cells in excreta from animals (Section 3.2) could be a significant source of these in wastewater or runoffs from agricultural fields.

### Fate of MTBC in wastewater

MTBC in wastewater could be affected by several processes, such as natural die-off and removal during wastewater treatment. This section addresses the impact of these processes on MTBC in the wastewater environment.

#### Survival of MTBC in excreta and wastewater

The survival of MTBC in wastewater has not been studied, however, several studies have investigated the survival of tubercle bacilli in other environments, such as soil, water, manure, feces and urine (Table 3). It was observed that tubercle bacilli inoculated in rivers at temperatures 8–12 °C and 15–20 °C can survive for 50 days [24]. Survival up to 6 months has also been reported for *M. tuberculosis* in water [67] and up to 41 months for *M. avium*, which is a common environmental mycobacteria [143]. There are also reports of the survival of this bacterium in water [67, 144, 145], using biodegradable organic material in the water especially in biofilms as carbon source [146]. The presence of these bacteria in urine could also provide further insights into their possible survival in wastewater [147]. The survival times of *M. bovis* and *M. tuberculosis* in human urine has been reported to be over 10 days at 4 °C and below 3 days at 22 °C [148]. In contrast, at 15 ^o^ C, mycobacteria have been reported to survive up to 6 weeks [54]. According to Scanlon and Quinn [147], the survival time of *M. tuberculosis* in sterilized manure kept at room temperature was up to 172 days. There are a few reports of extended survival for a year or more, generally in faeces or soil under optimal laboratory conditions. A study by Singh et al. [149] reported the survival of *M. tuberculosis* in faeces for 8 weeks or longer if protected from light. Table 3 gives examples of these studies on the survival of MTBC in different environments, showing the paucity of data for wastewater. The characteristics or complexity of wastewater differ significantly from water, feaces and urine, therefore the survival in these matrices may be different from wastewater. This warrants further research in understanding the survivability of MTBC in wastewater, especially considering the potential for GITB infections as a result of the ingestion of contaminated water and food.

**Table 3:** Reports on the survival of MTBC in different environmental matrix

Specific MTBC organism	Sample matrix	Survival period	References
*M. bovis*	Soil	150 days	[150]
*M. tuberculosis*, *M. bovis* and *M. canetti*	Soil	Survival of the distinct mycobacteria in the soil for 12 months	[151]
*M. tuberculosis*	Soil & water	persisted for 9 months	[67]
*M. bovis*		88 days in soil, 58 days in water and hay, and 43 days on corn	[81]
*M. bovis*	Manure	172 days	[152]
*M. bovis*	River water & distilled water	After 50 days, could still be cultured	[24, 153]
*M. bovis*	liquid manure	176 days	[147]
*M. bovis*	vegetables stored at -20 °C and 23 °C	112 days	[154]
*M. bovis*	Soil, urine and faeces	6 weeks	[52, 155]
*M. bovis*	wet soil	21 months	[156]

#### Factors affecting the survival of MTBC in wastewater

The survival of MTBC in different matrices could be influenced by several factors, such as temperature, moisture, pH, inhibitors and protection against solar radiation (ultra-violet) [77]. Intrinsically, MTBC cells can withstand desiccation due to the presence of a dense external cell wall composed of a large number of fatty acids [157]. For instance, *M. tuberculosis* was found to still be viable after exposure to high temperatures for several months [158]. Although the mechanisms responsible for this feature are not well-known, reports have indicated a possible role of endogenous synthesis of trehalose [157, 159]. Additionally, mycobacterial cells are known to be hydrophobic [41], which may result in their attachment to solid particles in the water environment. This could also play a role in the extensively reported biofilm formation by mycobacterial cells [135, 160–162]. Biofilm formation is a process that represents the most successful adaptation of bacteria against several environmental factors. It has become increasingly evident that biofilms in drinking water supply systems provide a transient or long-lasting habitat for many microbes, including human pathogens [95]. Biofilms provide protection against environmental stresses, e.g., desiccation, starvation and the presence of toxics [163, 164]. Coupled with their natural ability to withstand desiccation, the wastewater environment with high suspended solids enhancing biofilm formation could provide an additional layer of protection for MTBC cells, enhancing their survival. Mycobacteria are known to have a narrow pH range between 6.2 and 7.3 [165]. For instance, *M. tuberculosis* is reported to have extreme sensitivity to acid [165], but there have been reports of the intrinsic ability of some mycobacteria to maintain intra-cellular pH [166]. This gives *M. tuberculosis* the ability to survive in acidic wastewater conditions.

Additionally, microorganisms including *M. tuberculosis* have been reported to be amoeba-resistant which may enhance their survival in wastewater. *M. tuberculosis* [167] and *M. bovis* [168] could survive for hours to days in the amoebal trophozoites. The observation that *M. tuberculosis* and *M. bovis* organisms were engulfed by *Acanthamoeba polyphaga* trophozoites agreed with previous observations made when co-culturing *M. tuberculosis* organisms with the free-living amoeba *Dictyostelium discodium* [95, 169]. Mycobacteria survived in the cysts for up to 18 days and cysts protected *M. tuberculosis* organisms against mycobactericidals (5 mg/mL streptomycin and 2.5% glutaraldehyde). This data indicates that MTBC organisms are amoeba-resistant organisms, as previously demonstrated for non-tuberculous, environmental mycobacteria [95, 120, 170]. Inter-cystic survival of tuberculous mycobacteria, except for *M. canettii*, could therefore protect them against biocides and play a role in their survival [95, 151]. There is evidence to suggest that under starvation caused by nutrient limitations, low pH and lack of oxygen, a nonreplicating state is induced in some mycobacterial cells caused by the metabolic state of the pathogen [171]. Some MTBC organisms, like *M. avium,* can survive rapid shifts in oxygen content for prolonged periods by altering their metabolism from aerobic to anaerobic and vice versa [172].

Therefore, it is plausible that MTBC may be able to survive in wastewater, through both intrinsic (cell wall) and extrinsic factors (biofilms). However, the lack of information on the survival of MTBC in wastewater, as mentioned before, makes it difficult to conclusively determine the impact wastewater conditions may have on this group of organisms.

#### Removal of *Mycobacterium tuberculosis* complex during wastewater treatment

Wastewater treatment plants (WWTPs) serve as the guts of the population, receiving and digesting various human pathogens [125]. Several studies demonstrate that human pathogenic or opportunistic bacteria may survive treatment processes [173–175]. Radomski et al. [80] reported *Mycobacterium* concentrations of 5.5 × 10^5^ (±3.9 × 10^5^) copies/L in untreated wastewater and 0.74 × 10^4^ ± 1.40 × 10^4^ copies/L (in 7 positive samples among 13) detected in the final treated wastewater after decantation and biofiltration, and 1.04 × 10^6^ ± 1.75 × 10^6^ copies/g (in 3 positive samples among 6) in sludge. The most removal of mycobacteria (98.6 ± 2.7%, i.e. 2.4 ± 0.7 log_10_) was achieved by physical-chemical decantation (primary treatment) and the remaining mycobacteria were removed by biofiltration (secondary treatment) in this study. A study by Chandra and Arora [176], also reported 50% removal of mycobacterial load during primary sewage treatment processes.

Despite these reports of *M. tuberculosis* removal during wastewater treatment, there are contrasting reports where these organisms are reported to be detected more frequently in both the activated sludge and effluent, than the influent [177, 178]. Additionally, pathogenic *Mycobacterium* sp. have been reported in treated wastewater effluents from a WWTP treating salty wastewater [177, 179]. Da Silva et al. [180] investigated the microbial communities present in effluent samples from two independent field-scale swine WWTPs and concluded that *Mycobacteria* were abundantly observed in the final effluent. This is corroborated by Cai and Zhang [125], through metagenomic analysis, where a low abundance of the genus *Mycobacterium* was observed in the influent as compared to both the activated sludge and effluent. These reports indicate that wastewater treatment plants may have varying efficiencies in the removal of MTBC cells.

##### Impact of wastewater disinfection processes on MTBC

Tertiary treatment of wastewater usually involves the use of disinfection processes aimed at inactivating microbial organisms before discharge. These processes include chlorination, ozonation, and UV treatment [181–184]. Previous researchers have reported that several strains of mycobacteria are 100–330 times more resistant to chlorine than *E. coli* [185, 186], which is usually used as an indicator for wastewater treatment efficiency. Slow-growing mycobacteria are unaffected at the higher chlorine disinfection, confirming past reports of their high resistance to chlorination [178, 185]. Several, other studies have observed resistance of some mycobacteria to the normal chlorination process used either in drinking water or wastewater treatment plants [187, 188]. The peculiar structure of the mycobacterial cell wall skeleton partly explains the high resistance of mycobacteria to chlorination [189]. In mycobacteria, the peptidoglycan is covalently linked to mycolic acids, consisting of long fatty acids up to 90 carbon atoms, through an arabinogalactan bridge. Mycolic acids confer acid fastness to bacilli and represent a thick, hydrophobic barrier preventing diffusion and lowering permeability [189, 190]. Chen et al. [191] showed that the resistance of *Mycobacteria* to free chlorine was attributed to the cell membrane composition and observed that the richness of the long-chain saturated fatty acid or rareness of unsaturated fatty acid in the cell membrane might partly explain the higher chlorine resistance of *Mycobacteria* over other bacteria. The high concentration of mycolic acid and slow growth, adherence to surface and hydrophobicity of mycobacteria have been reported to be primarily responsible for the high resistance of mycobacteria to chemical disinfection [192, 193]. Comparatively, UV irradiation was more effective in eliminating *Mycobacterium,* however, Lee et al. [192] reported that mycobacteria are 2–10 times more resistant to UV than *E. coli*. Nevertheless, the absence of residual disinfection and low penetrability in water containing suspended solids are the major disadvantages of UV irradiation on a mass scale [193], especially in wastewater treatment.

## The potential risk of infection with *Mycobacterium tuberculosis* complex found in wastewater during wastewater treatment processes

The reported MTBC in treated and untreated wastewater could result in infections for different populations that may be exposed either directly or indirectly [99]. Direct exposure to wastewater could be a major route mainly for WWTP workers, farmers using the treated wastewater for irrigation and the general public exposed to either untreated wastewater within the community or effluent discharge from WWTPs. Despite this potential risk, there is a scarcity of studies in this regard. This section, therefore, discusses the potential of infection using information from related fields but not specifically for MTBC.

### Potential risks of infection for wastewater operators/workers

Most MTBC infections are usually through inhalation of aerosols or droplets, produced either through the coughing or sneezing of infected individuals [194]. Therefore, inhalation of water aerosols may represent the major route of exposure to MTBC in wastewater. Exposure through this pathway may expose three main groups of people: (1) individuals that shed viable pathogens into the toilet and are then exposed to these pathogens during the flush of the toilet, (2) individuals that come into contact with wastewater containing viable pathogens during the collection and treatment process, and (3) individuals that contact untreated wastewater containing pathogens during a spill or release of wastewater from the piping and collection system [195]. Liquid (droplet) aerosols notably are generated during wastewater aeration and also during the spray application of wastewater including sludge suspensions onto land. Aerosols generated during wastewater treatment might serve as a source of disease in wastewater treatment workers [196].

It is well known that exposure of wastewater treatment workers to bioaerosols carries a risk of negative health outcomes [197, 198]. This is based on the fact that sewage is known to contain a range of potential pathogens [173] and that some studies have suggested a correlation between exposure to WWTP bioaerosols and a range of respiratory and gastrointestinal symptoms [199, 200]. Occupation per se has not been considered as a determinant of contracting TB and its consequent morbidity. Sewage workers enter manholes and closed channels as part of their duties and also man the sewage treatment facilities. They work in confined spaces, closed channels and sewage treatment plants which employ technologies like up-flow anaerobic sludge blanket, activated sludge process, fluidized aerobic bioreactor, sedimentation, trickling filters, series of waste stabilization ponds which produce noxious fumes and bioaerosols [176, 201].

A study conducted by Chandra and Arora [202], consisting of 104 sewage workers with average occupational exposure to sewage work of 21.28 (±10.54) years. Approximately, 21% of the sewage workers had tuberculosis and 92.31% had at least one of the chronic respiratory diseases (COPD (Chronic obstructive pulmonary disease), Asthma or ACOS (Astha-COPD overlap syndrome)). It was concluded that sewage workers have an adverse chronic morbidity profile for tuberculosis. Therefore, there is an urgent need for epidemiological research and targeted screening and public health intervention for tuberculosis in sewage workers as an occupational group.

### Community infections from exposure to wastewater

Due to their small size and lightweight, particles are easily carried by wind and dispersed over considerable distances [203], which may cause infection in on-site workers as well as downwind residents. Several atmospheric factors, such as temperature, wind velocity, smog, and specific humidity, influence the aerosol spread as well as the ability of microorganisms to survive in the air [204, 205]. At very low humidity and high temperature, microbes face dehydration, whereas high humidity may give cells protection against solar radiation [206, 207]. The maximum distance for droplet transmission is currently unresolved, although pathogens transmitted by the droplet route have not been transmitted through the air over long distances [208]. It is likely that the distance droplets travel depends on the velocity and mechanism by which they are propelled from the source, the density of the secretions, environmental factors such as temperature and humidity, and the ability of the pathogen to maintain infectivity over that distance [208, 209]. Air microbiological analyses have commonly been conducted close to sewage treatment plants [206]. Concentrations of airborne bacteria varied in a wide range of 23–4878 CFU/m^3^ [210]. A study by Brenner et al. [211] recorded concentrations of 86–7143 bacterial CFU/m^3^ air at a distance of 25 m from the surface of an aeration basin well [206]. High microbial numbers were also reported in locations close to the WWTP [206].

In addition to aerosols generated during wastewater treatment, the reuse of wastewater for irrigation could also lead to the generation of aerosols [212, 213]. Aerosols generated during wastewater treatment and reuse are affected by the same factors as aerosols from the WWTPs. These processes could therefore be a significant route through which the general public may be exposed to MTBC in wastewater leading to infections. However, despite these potential risks, a few studies to date have focused on measuring the risks of infection with TB as a result of aerosols [104, 214] but no study has focussed on measuring the risks of infection with TB as a result of aerosols containing MTBC from WWTPs. This is, therefore, a research niche that requires further studies.

The detection of pathogenic mycobacteria in treated wastewater [177, 179] could potentially result in the contamination of surface water. Therefore, exposure to this contaminated surface water may result in infections. However, it is worth noting that the main route of transmission of TB is through aerosols, therefore the risks of infection from exposure to surface water may be low unless the exposure involves the generation and inhalation of these aerosols.

## Conclusion and recommendations

The reviewed literature showed that MTBC could potentially survive in wastewater for months, this could be attributed to their cell physiology and ecology. Additionally, although wastewater treatment has been shown to reduce the concentration of several bacteria, including these MTBC members, there are a significant number of reports on their occurrence in treated wastewater.

The possible exposure of WWTP workers to aerosols generated during wastewater treatment raises the potential risks for infection through this route. Several studies have shown the occurrence of pathogens in aerosols from WWTPs. Additionally, risks of infection could exist for the general public due to the transport of these aerosols further away from the WWTPs or to aerosols generated during wastewater reuse.

This review also exposed gaps in our knowledge on the occurrence and fate of MTBC in wastewater. This calls for further studies to address these areas,

1. Survival in wastewater: No study has explicitly looked at the survival of MTBC in wastewater and the factors influencing these. The conclusion drawn in this review on MTBC survival in wastewater was made based on survival data gathered for other environments like water and urine. Therefore, there is a need to determine their survival in wastewater under field conditions.
2. Risk reduction for sewage workers: The potential risks of infection for sewage workers due to exposure to aerosols requires the implementation of protective measures. Personal respiratory protection devices including the use of particulate respirators (N95 respirators or equivalent could potentially reduce or eliminate the risks of infection with MTBC through the inhalation of contaminated aerosols.
3. Risk assessment: There is the need for a further study to ascertain full pathogen (MTBC) occurrence and concentration in aerosols and determine the link with infections within the workers (occupational health study for WWTP workers)
4. Change in technology: It has been suggested that the use of diffused aeration technology results in a drastic reduction in the generation of aerosols. This could potentially eliminate the transmission of pathogens through aerosols. Alternatively, some researchers have theorized that it should be possible theoretically to reduce the size of the bubble for aeration so that eventually the resulting droplets and particles would be too small to carry any microorganisms.
5. Adherence to the distancing of settlements and WWTPs/wastewater reuse cites: Siting WWTPs and wastewater reuse irrigation sites away from residential areas could potentially reduce the exposure of the general public to aerosols generated during these processes.
6. There is also a need for an improvement on the methods of surveillance that are being used to track the prevalence of tuberculosis as it has been reported that there may be other potential sources of TB from the environment. There is a need for understanding the prevalence, and distribution of *Mycobacterium tuberculosis* complex organisms in the environment specifically wastewater. The prevalence of these tuberculosis-causing microorganisms in the untreated sewage may provide vital information in estimating not only the occurrence but also resistance in the associated population without clinical data on TB and its antibiotic resistance pattern.

## Data Availability

All reviewed articles, books or websites are included under the reference section.

## Abbreviations

AMB: Amphotericin B
COPD: Chronic obstructive pulmonary disease
ACOS: Asthma-COPD overlap syndrome
CPC: Cetylpyridinium chloride
CFU: Colony forming units
GI: Gastrointestinal
GITB: Gastrointestinal Tuberculosis
HIV: Human immunodeficiency virus
HIV/AIDS: Human immunodeficiency virus/ acquired immunodeficiency syndrome
MDR-TB: Multidrug-resistant *M. tuberculosis*
MTBC: *Mycobacterium tuberculosis* complex
NAL: Nalidixic acid
PACT: Polymyxin B, Amphotericin B, Carbenicillin and Trimethoprim
PANTA: Polymyxin-B, Amphotericin-B, Nalidixic acid, Trimethoprim, Azilocillin
PCR: Polymerase chain reaction
RT: Reverse transcriptase
TB: Tuberculosis
VAN: Vancomycin
VBNC: Viable but nonculturable
WGS: Whole-genome sequencing
WWTPs: Wastewater treatment plants
